# Predicting bioavailability change of complex chemical mixtures in contaminated soils using visible and near-infrared spectroscopy and random forest regression

**DOI:** 10.1038/s41598-019-41161-w

**Published:** 2019-03-14

**Authors:** S. Cipullo, S. Nawar, A. M. Mouazen, P. Campo-Moreno, F. Coulon

**Affiliations:** 1 Cranfield University, School of Water, Energy and Environment, Cranfield, MK430AL UK; 2 Department of Environment, Ghent University, Coupure 653, 9000 Gent, Belgium

## Abstract

A number of studies have shown that visible and near infrared spectroscopy (VIS-NIRS) offers a rapid on-site measurement tool for the determination of total contaminant concentration of petroleum hydrocarbons compounds (PHC), heavy metals and metalloids (HM) in soil. However none of them have yet assessed the feasibility of using VIS-NIRS coupled to random forest (RF) regression for determining both the total and bioavailable concentrations of complex chemical mixtures. Results showed that the predictions of the total concentrations of polycyclic aromatic hydrocarbons (PAH), PHC, and alkanes (ALK) were very good, good and fair, and in contrast, the predictions of the bioavailable concentrations of the PAH and PHC were only fair, and poor for ALK. A large number of trace elements, mainly lead (Pb), aluminium (Al), nickel (Ni), chromium (Cr), cadmium (Cd), iron (Fe) and zinc (Zn) were predicted with very good or good accuracy. The prediction results of the total HMs were also better than those of the bioavailable concentrations. Overall, the results demonstrate that VIS-NIR DRS coupled to RF is a promising rapid measurement tool to inform both the distribution and bioavailability of complex chemical mixtures without the need of collecting soil samples and lengthy extraction for further analysis.

## Introduction

A number of anthropogenic activities such as waste disposal, mining activities, manufacturing, and petrochemical industries as well as poor environmental management practices have left a legacy of contaminated sites across Europe and worldwide^1^. Contaminants of concerns are often present on site as a complex mixture^2^ and their co-occurrence and interactions can impact their adsorption behaviour in soil, and influence their availability^3^. Recovery of brownfield sites is often challenging as hazards are very heterogeneous, reliable exposure data are lacking, and remediation often requires large investments and involves multiple stakeholders^4^. Risk assessment is recognised as a robust process to support decision-making strategies for contaminated land, and to prevent further damage to the environment and human health^5^. It has been further shown that measuring only the total concentration of contaminants in soil does not give a useful basis for the evaluation of the potential risks to human and the Environment^6^. In fact, in the United Kingdom, and increasingly across the world, over the last decade the end-point of remedial activity is defined by the concentration of the chemicals of concern likely to pose significant risk, the bioavailable concentration^7,8^. Moreover, contaminants bioavailable fraction is highly dependent on contaminant chemical properties as well as soil physicochemical properties^9^. Similarly, several risk-based frameworks for contaminated soils have been published under the auspices of national and international regulatory organizations each reflecting national legislation, a range of expert judgments and socioeconomic issues^10^. However they all typically adopt a three tiered approach with increasingly sophisticated levels of data collection and analysis as an assessor moves through the tiers. The common steps include (1) developing a conceptual site model (CSM) of the site based on a-priori information and historical land use, (2) conducting a preliminary site assessment to refine the initial CSM, (3) deciding if further assessment (generic and detailed) are needed. Risk assessments generally require more data when moving from preliminary to generic (comparison with general contamination threshold) and to detailed risk assessments (comparison with site-specific contamination threshold). Therefore, in order to establish practical and sustainable criteria to achieve a reasonable level of clean-up for the intended land use, it is important to: (1) reduce uncertainties associated with sampling especially for large site, (2) deliver cost-effective approaches to support site investigation, (3) reduce analytical cost associated with complex-contaminant assessment, and (4) reduce significantly the time associated with sampling and subsequent laboratory analysis.

The preliminary site investigation plays a key role in the risk assessment process, as the accuracy of the information gathered at this step is fundamental to correctly manage the associated time and costs^11^. Often, at this stage, sample collection is not included, and probability-based sampling strategies are mostly designed from conceptual site model information, combining random and selected sampling starting points^12^. In this regards rapid-measurement tools (RMT), such as reflectance spectroscopy, including visible and near-infrared (VIS-NIR) or mid-infrared (MIR) spectroscopy, can support the decision making strategies, by improving quality and quantity of information collected during site investigation^13^. Additionally, the on-the-go instrument could be used to perform real-time monitoring and assessing on-site remediation efficacy or natural attenuation^14^.

The reflectance spectra of contaminated soils in the visible near-infrared and short wave infrared region (400–2500 nm) (VIS-NIR-SWIR) allows rapid and cost-effective acquisition of soil information based on the unique absorption spectra of specific chemical compounds^15,16^. VIS-NIRS has been successfully used to estimate both petroleum-derived compounds^15^ and heavy metals^17^ in genuine and spiked soil samples. In particular, VIS-NIRS coupled with RF modelling has been previously shown to outperform other regression techniques such as partial least square regression (PLSR) as it is able to account for the non-linearity associated with the soil spectral responses^13^.

The principle of VIS-NIRS is based on the frequencies of which molecules rotates or vibrates generating discrete measurable energy levels^12^. Infrared spectroscopy is mostly used for the estimation of organic compounds which allows the determination of a fixed-wavelength responding to the vibration caused by C-H and C-C bonds stretching and bending^18^. However, Wu *et al*.^19^ showed while there is no direct spectral response of HM within the NIR range, VIS-NIRS can detect HM due to vibrations of -OH bonds as a result of their association with Fe oxides, clays and organic matter. Therefore most of the trace elements can be easily detected at very high concentrations (i.e. Cr and Cu at >4000 mg/kg;^20^ and with reasonable accuracy at low levels^14^.

In the past five years, several studies have shown that VIS-NIR can successfully predict in soil both total concentration of HM^12,21–24^ and total concentration of PHC^13,25^. However none have yet investigated the feasibility of using VIS-NIR as a RMT to predict on site the bioavailable concentration of HM and PHC, simultaneously.

In this study, the performance of VIS-NIR spectroscopy coupled to RF regression was therefore assessed for predicting the total and the bioavailable concentrations of heavy metals/metalloids and petroleum hydrocarbons mixtures in five genuinely-contaminated soils.

## Materials and Methods

### Sample collection and preparation

**Figure 1:**
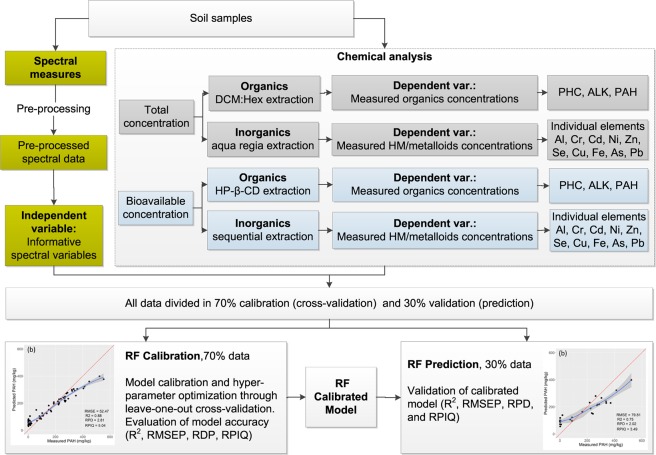
Illustrative block diagram showing the different steps for the estimation of complex chemical mixtures of total and bioavailable concentrations in soils using chemical methods and VIS-NIR coupled with Random Forest (RF). DCM: dichloromethane; Hex: hexane; HP-β-CD: hydroxypropyl-β-cyclodextrin; PHC: Petroleum hydrocarbons; HM: heavy metals; PAH: polycyclic aromatic hydrocarbons; ALK: Alkanes; Al: aluminium; Cr: Chromium, Cd: Cadmium; Ni: Nickel, Zn: Zinc; Se: Selenium, Cu: Copper; Fe: Iron; As: Arsenic; Pb:Lead, ML: Machine Learning,; LOOCV: leave-one-out-cross-validation; R2:coefficient of determination; RMSEP: root mean square error of prediction; RPD: ratio of prediction deviation; RPIQ: ratio of the performance to interquartile distance.

Three genuinely contaminated soils, denoted as Soil 1, Soil 2, and Soil 3, were collected from a treatment site located in the United Kingdom. Two additional soil types were collected from a rural site contaminated by diesel (Soil 4), and mineral oil (Soil 5). Information regarding original location of the soil samples collected, and specific details regarding the treatment applied, were not disclosed by the treatment facility to maintain anonymity and confidentiality. More details of the soil physicochemical characteristics are provided and discussed in the Supplementary Material (Table S1). All samples were collected randomly from the soil layer down to a depth of 30 cm and immediately stored at 4 °C to minimise biological transformation and other chemical reactions. A total of 21 samples were collected for each soil type (e.g., for the 5 soil types a total of 105 samples) and split into five sub-samples; one of them was used for spectroscopic measurements and the other four for chemical analytical determinations of total and bioavailable (HM/metalloids and PHC) contents. An outline of experimental and analytical procedures used is presented in Fig. 1.

### Extraction and quantification of total and bioavailable petroleum hydrocarbons and heavy metals and metalloids

The method used to determine total petroleum hydrocarbons, including PAH and ALK fractions in soil, was adapted from the procedure described by Risdon *et al*.^26^. Briefly, PHC were extracted using solvent ultra-sonication from 2.5 g of soil mixed with 15 mL of a mixture of 1:1 dichloromethane: hexane. The bioavailable hydrocarbons content was instead extracted using 20 ml of a 50 mM solution of hydroxypropyl-β-cyclodextrin (HP-β-CD) as described by Cipullo *et al*.^27^. Extraction, identification, and quantification of total and bioavailable PHC, PAH, and ALK were performed by gas chromatography-mass spectrometry (GC-MS) as described by Cipullo *et al*.^28^.

The pseudo-total element digestion was performed according to the ISO 11047 method with aqua regia^29^. The bioavailable heavy metals and metalloids content were determined using a modified procedure of the sequential extraction method of Cave *et al*.^30^. Briefly soil samples (2 g) were consecutively extracted by addition of 10 mL of a nitric acid solution of increasing concentration from 0 to 5 M. All total and sequential extracts were analysed by inductively coupled plasma mass spectrometry (ICP-MS A NexION^®^ 350D ICP-MS, Perkin Elmer) as described by Cipullo *et al*.^28^. In this work the HM bioavailable fraction was considered to be the amount of elements associated with pore water phase (readily available or bioavailable), and carbonates phases (potentially available with time).

### Soil spectra analysis

#### Spectra collection

Soil samples were air-dried and sieved (2 mm) to get the fine earth and separate large particles like plant parts (roots, stem, and leave), cobbles, and pebbles^31^. The fine earth was mixed well, before three sub-samples were made from each soil sample and packed into three plastic Petri dishes (1 cm height, and 5.6 cm in diameter). The sample surface was smoothened gently with a spatula to obtain optimal diffuse reflection, and hence, a good signal-to-noise ratio^32^. The diffuse reflectance spectra of the soil samples were measured using an ASD LabSpec2500^®^ VIS–NIR spectrophotometer (350–2500 nm). The spectral resolution varied from 3 nm in 700 nm and 6 nm in 1400–1200 nm (Analytical Spectral Devices Inc., CO, USA). A high-intensity probe that has a built-in light source made of a quartz-halogen bulb of 2727 °K was placed in contact with soil sample to collect the spectra. Measurement was done under dark conditions, to control the artificial illumination and reduce the effects of stray light. Before scanning the ASD instrument was first warmed-up for at least 30 min, and then calibrated by a white Spectralon disc of almost 99% reflectance. For each sample, 3 successive spectra were acquired at three equidistant positions approximately 120° apart and these were averaged in one representative spectrum of a soil sample. Representative diffuse reflectance spectra of the five soil samples analysed are shown in Supplementary Information in Fig. S1.

#### Spectra pre-treatment

The raw average spectra of the 105 samples were subjected to pre-treatment including successively, noise cut, maximum normalization, first derivative and smoothing using *prospectr-R package*^33,34^ in RStudio (Version 1.1.423 – ^©^ 2009–2018 RStudio, Inc.). Maximum normalisation was implemented to align all spectra to the same scale and to obtain even distribution of the variances and average values. Spectra were then subjected to first derivation using Gap–segment derivative (gapDer) algorithm^35^ with a second-order polynomial approximation. Finally, the Savitzky-Golay (SG) algorithm with a window size of 11 and polynomial of order 2 was carried out to remove noise from spectra^36^.

### Random forest regression analysis

#### Selection of Input variables

A two-dimensional data matrix was created by combining the reference values of chemical analyses of PHC, PAH, ALK, and HM/metalloids contents (dependent variables) and pre-treated spectra (independent variables) of 105 soil samples. Removal of outliers for each data set was based on principal components analysis (PCA). PCA was followed by randomly splitting the dataset into 70% for calibration (74 samples) and 30% for prediction (31 samples). PCA can be used to obtain a qualitative Vis-NIR discrimination of the information contained in the soil spectra (350–2500 nm)^37^. This multivariate technique can be used to reduce the dimensionality of large data sets^38^. The principal components (PC) identified were then plotted to investigate the relationships among data, as well as identifying similarities or patterns. Furthermore the PC were used to investigate wavebands typically associated with presence of contaminants such hydrocarbons or heavy metals^16^.

#### Model calibration

The hyper-parameter optimisation and calibration of the model was done through leave-one-out cross-validation (LOOCV)^39^. For the calibration dataset of n = 74 samples, LOOCV means that n-1 samples are used to calibrate the model and 1 sample is used to assess the accuracy; this is repeated n times for each single sample in the calibration dataset^40^. Model accuracy (predicted vs measured PHC, PAH, ALK and HM contents) was evaluated using the coefficient of determination (r^2^), the root mean square error of prediction (RMSEP), the ratio of prediction deviation (RPD) (standard deviation of measured values divided by RMSEP) and the ratio of the performance to interquartile distance (RPIQ). In general, a good model prediction should correspond to high r^2^, RPD and RPIQ, and low RMSEP values. In particular, model classification criterion adopted in this study were based on RPD values, which were divided into six classes: of excellent (RPD > 2.5), very good (RPD = 2.5–2.0), good (RPD = 2.0–1.8), fair (RPD = 1.8–1.4), poor (RPD = 1.4–1.0), and very poor model (RPD < 1.0)^41^. The model hyper-parameters optimised during the LOOCV are the number of trees to be grown (*ntree*), number of predictor variables used to split the nodes at each partitioning (*mtry*), and the minimum size of the leaf (*node size*). The hyper-parameter optimization returned ntree = 500, mtry = 2 and note size = 3. All PHC, PAH, ALK, and HM models of both the total and bioavailable contents were developed with Random Forest-R package^42^, utilising the Breiman and Cutler’s Fortran code^43^.

#### Prediction

The calibrated models were then validated using the prediction data sets (31 samples) for both the total and bioavailable contents of PHC, PAH, ALK and each individual HM. Once again the accuracy of the prediction (predicted vs measured) was evaluated by r^2^, RMSEP, RPD, RMSEP, and RPIQ and the outcome classified according to the criteria of Viscarra *et al*.^41^ as described above.

## Results and Discussions

### Total and bioavailable PHC and HM contents in soils

**Figure 2:**
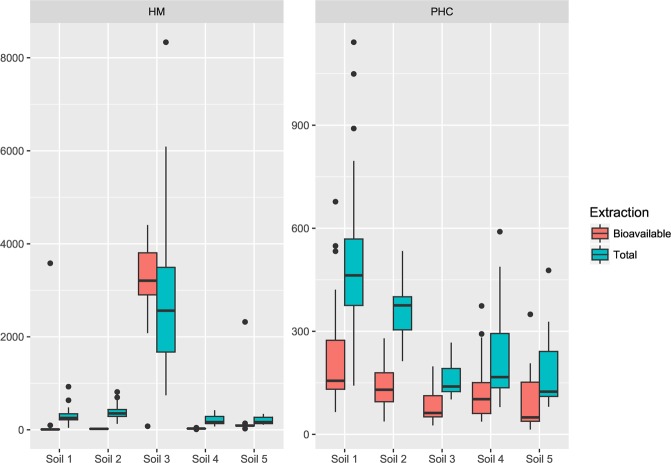
Box plot representing total and bioavailable concentrations (mg/kg) of heavy metals/metalloids (HM) (left) and petroleum hydrocarbons (PHC) (right) across the five soil types (n = 105). Black dots represent outlier samples.

The industrial soils (Soil l and 2) had the highest concentrations of total PHC with average of 445 mg/kg of which about 40% was found to be bioavailable (Fig. 2). The PHC distribution was dominated by the EC_21.35_ PAH fraction which represented between 45% and 55% of the total PHC. The dominant ALK were within the EC_16–35_ fraction. These profiles are typical of aged contamination. The average HM content for both soils was 350 mg/kg and the bioavailable content was low (<30%) especially for Al, Zn, Fe and Pb.

The other industrial contaminated soil (Soil 3) had a concentration of HM 8 times higher (2800 mg/kg) and the PHC concentration was 3 times lower than Soil 1 and 2 (Fig. 2). The EC_21–35_ PAH fraction contributes over 20% of the total PHC content. In contrast the bioavailable concentration were high for Zn and Pb (≥90%), low for Cu, Ni, Se and Cd (29, 34, 33, and 67%), and very low for Al (6%), Fe (1%) and Cr (3%) (data not shown).

In the rural contaminated soils (Soil 4 and 5) the total average PHC content was two times lower compared to the industrial soils ranging between 230 and 180 mg/kg, of which about 50% was found to be bioavailable (Fig. 2). As per the contaminated industrial soils, the PHC distribution was dominated by the EC_21–35_ PAH fraction and the ALK fraction EC_16–35_. The total HM (<200 mg/kg) were also 2 times lower than those found in the industrial contaminated soils (Fig. 2). The average bioavailable concentrations of metals for rural soil samples were high for Cd only (≥90%), low for Zn, Cu, Ni and Se (24%, 38%, 16% and 12%), and very low for Al, Fe, Pb and Cr <(1%) in Soil 4. In Soil 5 HM were more available, in particular concentrations were high for Cu, Se, Cd and Pb (≥90%), low for Zn and Ni (66% and 43%), and very low for Al (8%), Fe (6%) and Cr (2%) in Soil 5.

### Model calibration and performance

Data obtained from soil spectral analysis and chemical analysis (total and bioavailable PHC, PAH, ALK and HM concentrations) were used in the calibration of the RF regression model; descriptive statistics of data used at this step are provided in Table 1. The results of LOOCV of the ML model for total and bioavailability organic compounds are shown in Table 2 and Fig. S2, *Supplementary Information*. The LOOCV results for both the total (r^2^ = 0.88, RPD = 2.81, RPIQ = 5.04, and RMSEcv = 52.47 mg/kg) and bioavailable (r^2^ = 0.82, RPD = 2.38, RPIQ = 3.62, and RMSEcv = 33.62) PAH were better than those for total and bioavailable PHC and ALK (Table 2 and Fig. S2). The lowest accuracy was observed for ALK; however the LOOCV results of the total concentration were slightly better than those of the bioavailable concentration; r^2^, RPD, RPIQ, and RMSEcv values of 0.82 and 0.77, 2.42 and 2.10, 1.75 and 1.62, and 30.74 and 18.74 mg/kg, respectively (Table 2).

**Table 1:** Descriptive statistics of the calibration datasets of total and bioavailable contents of PHC, PAH, ALK and HM/metalloids used for the RF modelling.

			No	Min	1^st^ Q	Median	Mean	3^rd^ Q	Max	SD
Organics	Total (mg/kg)	PHC	74	79	137	241	285	389	1049	188
		PAH	73	0.3	2.1	102	145	267	553	160
		ALK	73	49	109	126	146	163	496	74
	Bioavailable (mg/kg)	PHC	73	14	48	109	127	159	548	107
		PAH	73	0.2	1.2	60	76	131	326	82
		ALK	73	7.3	32	47	55	62	263	39
Inorganics	Total (mg/kg)	Al	74	2375	7289	12301	14409	18808	46195	9605
		Cr	73	5	17	25	29	37	85	16
		Cd	72	0.1	0.2	0.3	0.4	0.6	2	0.4
		Ni	74	2	11	15	18	26	49	10
		Zn	73	15	64	108	244	164	1964	393
		Se	72	0.4	1	2	2	3	6	1
		Cu	73	4	12	27	33	40	128	25
		Fe	74	787	10857	15300	17969	20955	57669	10822
		As	73	1	7	10	11	13	34	6
		Pb	74	9	31	61	288	131	2864	600
	Bioavailable (mg/kg)	Al	72	1	8	234	339	685	1037	355
		Cr	73	0.1	0.3	1	1	1	2	1
		Cd	73	0.1	0.2	0.2	0.3	0.2	2	0.4
		Ni	74	1	1	3	3	4	12	2
		Zn	72	4	9	15	314	26	1911	624
		Se	72	0.1	0.5	1	1	1	2	0.4
		Cu	72	0.2	2	6	7	12	18	6
		Fe	73	5	8	98	171	159	928	244
		As	72	0.3	0.5	1	1	1	1	0.2
		Pb	74	0.1	0.3	5	295	54	2463	690

**Table 2:** RF outputs for the calibration of the total and bioavailable concentrations of PHC, PAH, ALK and HM/metalloids in the contaminated soil samples.

		Compound	N°	R^2^	RMSE (mg/kg)	RPD	RPIQ
Organics	Total (mg/kg)	PHC	74	0.83	78.2	2.4	3.2
		PAH	73	0.88	52.5	2.8	5.1
		ALK	74	0.82	30.7	2.4	1.8
	Bioavailable (mg/kg)	PHC	74	0.80	48.5	2.3	2.5
		PAH	73	0.82	33.6	2.4	3.6
		ALK	74	0.77	18.7	2.1	1.6
Inorganics	Total (mg/kg)	Al	73	0.93	2195	4.1	5.2
		Cr	73	0.93	4	3.7	4.8
		Cd	72	0.92	0.1	3.5	5.2
		Ni	74	0.92	3	3.6	5.6
		Zn	73	0.9	121	3.3	1.8
		Se	72	0.88	0.4	3	4.2
		Cu	73	0.9	8	3.3	3.5
		Fe	74	0.92	2967	3.6	3.4
		As	73	0.89	2	3.1	3.2
		Pb	74	0.88	198	3	2.6
	Bioavailable (mg/kg)	Al	72	0.92	97	3.8	5
		Cr	73	0.92	0.1	3.7	5.3
		Cd	73	0.91	0.1	3.3	3.4
		Ni	74	0.77	0.9	3.1	3.6
		Zn	72	0.82	258	2.4	1.3
		Se	72	0.86	0.1	2.7	3.2
		Cu	72	0.89	1.5	3.7	6.5
		Fe	73	0.89	78	3.1	1.9
		As	72	0.86	0.07	2.8	3.1
		Pb	74	0.86	199	2.8	2.8

As for the organics, the LOOCV results for HM were better for the total than for the bioavailable concentration. Descriptive statistics of HM concentrations used in calibration step are presented in Table 1, and parameters used to establish goodness of the model are presented in Table 2 and Fig. S3 *Supplementary Information*. The highest LOOCV performance for the total concentration was obtained for Al (r^2^ = 0.93, RPD = 4.05, RPIQ = 5.17, and RMSEcv = 2194.5 mg/kg) followed by Cr, Fe, Ni, and Cd, whereas the worst performance is obtained for Se (r^2^ = 0.88, RPD = 2.99, RPIQ = 4.16, and RMSEcv = 0.36 mg/kg), followed by Pb, As, Zn and Cu (Table 2 and Fig. S3). The models developed for the bioavailable concentration showed some similarities to those of the total concentrations, for the calibration model. Again Al model for bioavailable concentration was the highest performing in LOOCV (r^2^ = 0.92, RPD = 3.77, RPIQ = 4.99, and RMSEcv = 96.67 mg/kg), followed by Cr, Cu, Cd and Fe, whereas the lowest performance was obtained for the Zn model (r^2^ = 0.82, RPD = 2.41, RPIQ = 1.3, and RMSEcv = 257.87 mg/kg), followed by Se, As, Pb, Ni (Table 2 and Fig. S3).

### Model prediction: Estimation of total and bioavailable concentrations of complex chemical mixtures using RF regression

**Figure 3:**
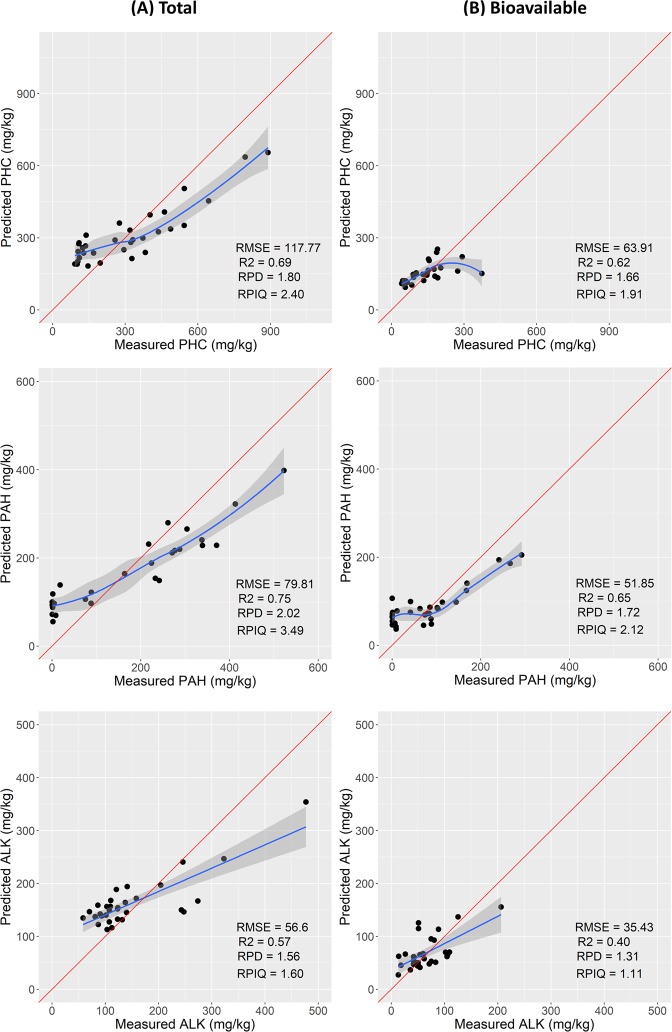
Scatter plots of the prediction datasets of total (**A**) and bioavailable (**B**) total petroleum hydrocarbons (PHC), aromatic (PAH) and alkanes (ALK), respectively.

**Figure 4:**
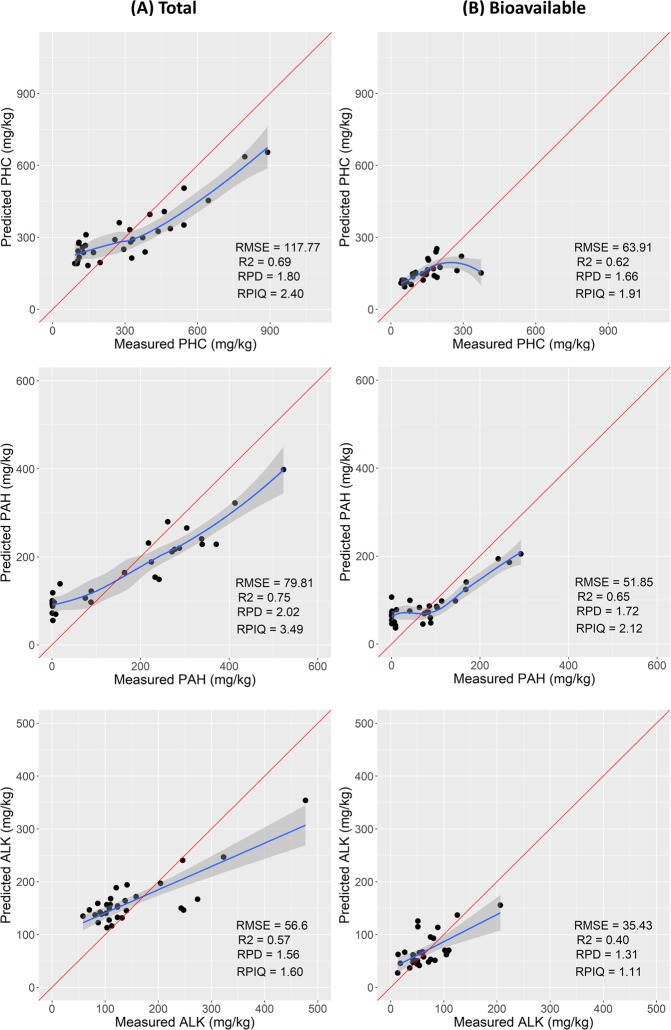
Scatter plots of the prediction datasets of total (**A**) and bioavailable (**B**) contents of HM/metalloids

The RF calibration model developed was further validated using the prediction sets (30% of the data) of total and bioavailable complex chemical mixtures concentration. The descriptive statistics are provided in Table 3. The models used for total and bioavailable prediction of both organic and inorganic compounds have the limitation of overestimating low values and underestimating high values (Figs 3 and 4). This trend has been previously observed in other studies^36,44,45^ and is associated with the RF regression model. The model response (output) is computed as the average (mean) of all of the trees in the forest, and the available values (measured points) constitute the pool from which the output is computed. For this reason, it is not possible to predict (estimate) values larger than the measured high-values.

**Table 3:** Descriptive statistics of the prediction datasets of total and bioavailable PHC, PAH, ALK and HM/metalloids used for the RF modelling.

		Compound	N°	Min	1^st^ Q	Median	Mean	3^rd^ Q	Max	SD
Organics	Total (mg/kg)	PHC	31	92	127	285	308	411	890	210
		PAH	31	0.6	2.9	190	172	285	522	160
		ALK	31	58	102	120	149	150	477	88
	Bioavailable (mg/kg)	PHC	31	42	58	130	131	177	374	80
		PAH	31	0.3	3.8	70	76	102	292	81
		ALK	31	35	13	54	65	82	206	38
Inorganics	Total (mg/kg)	Al	31	1543	5222	8920	12677	18772	33055	9113
		Cr	31	3	11	18	23	31	59	15
		Cd	31	0.1	0.1	0.2	0.4	0.4	1	0.4
		Ni	31	4	9	14	16	22	36	10
		Zn	31	30	66	105	303	320	1827	446
		Se	31	1	1	2	2	3	4	1
		Cu	31	6	12	21	27	25	103	23
		Fe	31	1109	5647	11825	15774	21352	40529	11672
		As	31	3	8	10	13	17	25	6
		Pb	31	11	40	106	314	291	2349	519
	Bioavailable (mg/kg)	Al	31	1	2	263	344	603	906	329
		Cr	31	0.1	0.4	1	1	1	1	0.4
		Cd	31	0.1	0.2	0.2	1	1	2	1
		Ni	31	1	2	3	4	5	8	2
		Zn	31	5	12	18	147	24	1176	343
		Se	31	0.2	1	1	1	1	1	0.4
		Cu	31	0.3	3	5	7	13	18	5
		Fe	31	6	18	142	252	426	816	290
		As	31	0.4	1	1	1	1	1	0
		Pb	31	0.1	0.3	5	577	1511	2408	888

#### Prediction of total and bioavailable PHC

Based on the Viscarra *et al*.^41^ classification of RPD classes, the RF prediction performance trend for the total and bioavailable concentrations was PAH > PHC > ALK very good and fair for total and bioavailable PAH, good and fair for total and bioavailable PHC and fair and poor for total and bioavailable ALK (Table 4). The prediction of the total concentration of PAH was of better performance (r^2^ = 0.75, RPD = 2.02, RPIQ = 3.49, and RMSEP = 79.81 mg/kg) than that for the bioavailable concentration (r^2^ = 0.65, RPD = 1.72, RPIQ = 2.12, and RMSEP = 51.85 mg/kg) (Table 4 and Fig. 3). Our prediction results are slightly better than the results reported by Douglas *et al*.^13^ for total PAH (r^2^ = 0.71, RPD = 1.99, and RMSEP = 0.99 mg/kg), and comparable to those results reported by Okparanma *et al*.^46^ using partial least squares regression (PLSR) for oil contaminated soil samples collected from the Niger delta, Nigeria. The difference of results can be attributed to variation in the concentration range as well as the standard deviation (SD) between our study (range from 0.30 to 533 mg/kg, SD of 160) and those reported by Douglas *et al*.^13^ (range from 0.52 to 312.28 mg/kg, SD = 40.20). Statistical similarity between the calibration and prediction sets including the range as well as SD can be observed indicating positive impact of the models performance^47^.

**Table 4:** RF outputs for the prediction for total and bioavailable concentrations of PHC, PAH, ALK and HM in contaminated soils.

		Compound	N°	R^2^	RMSE (mg/kg)	RPD	RPIQ
Organics	Total (mg/kg)	PHC	31	0.69	117.8	1.8	2.4
		PAH	31	0.75	79.8	2.0	3.5
		ALK	31	0.57	56.6	1.6	1.6
	Bioavailable (mg/kg)	PHC	31	0.62	63.9	1.7	1.9
		PAH	31	0.65	51.9	1.7	2.1
		ALK	31	0.40	35.4	1.3	1.1
Inorganics	Total (mg/kg)	Al	31	0.79	4101	2.2	3.3
		Cr	31	0.76	7	2.1	2.6
		Cd	31	0.76	0.2	2.1	2.3
		Ni	31	0.77	5	2.1	2.8
		Zn	31	0.71	235	1.9	1.7
		Se	31	0.67	0.6	1.8	2.9
		Cu	31	0.6	15	1.6	1.9
		Fe	31	0.72	5997	1.9	2.6
		As	31	0.72	3	1.9	2.8
		Pb	31	0.81	217	2.4	2.3
	Bioavailable (mg/kg)	Al	31	0.77	154	2.1	3.9
		Cr	31	0.75	0.2	2.0	3.4
		Cd	31	0.76	0.2	2.0	2.2
		Ni	31	0.65	1.3	1.7	2.3
		Zn	31	0.56	222	1.6	1.2
		Se	31	0.5	0.2	1.4	1.6
		Cu	31	0.6	3	1.6	3
		Fe	31	0.58	183	1.6	2.2
		As	31	0.45	0.2	1.4	1.7
		Pb	31	0.75	343	2.1	2.1

#### Prediction of total and bioavailable HM/metalloids

Results of the prediction set (for 31 samples) for HM/metalloids total concentration are rated as follow Pb > Al > Ni > Cr > Cd, where the highest performance was obtained for Pb (r^2^ = 0.81, RPD = 2.35, RPIQ = 2.30, and RMSEP = 216.62 mg/kg). The lowest prediction performance is obtained for Cu (r^2^ = 0.60, RPD = 1.59, RPIQ = 1.93, and RMSEP = 14.54 mg/kg), followed by Se, Zn, As and Fe (Table 4 and Fig. 4). On the basis of the RPD values, predictions of the total content of Pb (RPD = 2.35) was the best, and can be classified as very good, as well as the prediction of Al, Ni, Cr, and Cd with RPD values of 2.21, 2.13, 2.10, 2.10, respectively; whereas the prediction of Fe, As, and Zn can be classified as good with RPD values of 1.95, 1.92, 1.89, respectively. The Se and Cu can be classified as fair predictions with RPD values of 1.77 and 1.59, respectively.

The prediction models developed for the bioavailable concentration showed the highest performance for Al (r^2^ = 0.77, RPD = 2.13, RPIQ = 3.89, and RMSEP = 154.22 mg/kg), followed by Pb, Cr, Cd, and Ni, whereas the worst prediction was for As (r^2^ = 0.45, RPD = 1.37, RPIQ = 1.74, and RMSEP = 0.15 mg/kg), followed by Se, Zn, Fe, and Cu (Table 4 and Fig. 4). The prediction of the bioavailable concentrations shows differences of prediction quality, where Al, Pb, Cr, and Cd predictions are classified as very good with RPD values of 2.13, 2.10, 2.05, and 2.05, respectively. The prediction of Ni, Cu, Fe, Zn and Se can be classified as fair with RPD values of 1.73, 1.63, 1.58, 1.55, and 1.44, respectively, whereas As prediction is of the worst accuracy (RPD = 1.37) and can be classified as poor. It can be confirmed that Al and Pb models showed the highest prediction performance for both the total and bioavailable concentrations, but with relatively high RMSEP values of 4101.3, and 154.2 mg/kg for Al, and 216.6 and 343.1 mg/kg for Pb, for total and bioavailable concentration, respectively.

### Applicability of VIS–NIRS to predict bioavailability of complex chemical mixtures

Although there are to date no other studies that used VIS-NIRS to predict bioavailable concentrations of complex chemical mixtures of hydrocarbons and HM in soils, some comparison can be drawn with previous studies. For example, Cave *et al*.^48^ showed that PAH bioaccessibility in soil samples can be successfully predicted using a combination of soil properties (measured by NIR and MIR spectra) and physico-chemical properties of the PAH. The accuracy (measured vs predicted BPF) of the RF model used in this study was found to be good (RMSEP = 0.038 mg/kg) and precise (normalised RMSEP <15%). This confirms our findings that RF models which use infrared techniques in combination with organic contaminants and soil physico-chemical properties can be used to predict bioaccessible and bioavailable fractions with reasonable accuracy and precision.

Similarly, Chodak *et al*.^49^ used VIS–NIRS coupled with PLSR to determine the total and exchangeable concentrations of Zn and Pb in forest soil samples. However PLSR was found to be unsatisfactory for the prediction of both the total and exchangeable concentrations due to low RPD values (ranging between <1.3) and a tendency of underestimating both the total and the exchangeable HM at high concentrations. In contrast in our study, both the r^2^ and RPD values for the bioavailable HM were much higher (Zn r^2^ = 0.56 and RPD = 1.6; Pb r^2^ = 0.75 and RDP = 1.6; average for all HM r^2^ = 0.64 and RPD = 1.75) indicating that the RF model was better at predicting Pb bioavailable concentrations.

In another study, Li *et al*.^50^ showed a good prediction for the determination of metal ions in water samples using a pre-concentration step on a high capacity adsorbent material followed by NIR diffuse reflectance spectroscopy analysis. The r^2^ values of the PLSR model were 0.92, 0.96, and 0.99 for Hg, Pb, and Cd, respectively. These values are higher than the one obtained in our study (r ^2^ = n.a (Hg), r ^2^ = 0.75 (Pb), r ^2^ = 0.76 (Cd)). This could be attributed to (1) the use of a sorbent material rather than soil samples, (2) the homogeneous range of concentration obtained in the pre-concentration step where elements were taken up from the aqueous solutions and transferred to the high capacity adsorbent (concentration range Hg = 4.3–50.4 mg/l, Pb = 4.93–48.8 mg/l and Cd = 5.9–48.8 mg/l). In contrast in our study genuine contaminated soil samples from 5 different locations have been used, creating a more heterogeneous dataset with different soil characteristics and different concentrations (Hg below detection limit; Pb = 0.03–2463.4 mg/kg; Cd = 0.03–6.79 mg/kg).

## Conclusion

This study demonstrated that VIS-NIRS can be used as a rapid measurement tool for discriminating and estimating complex chemical mixtures of heavy metals, metalloids and petroleum hydrocarbons in soils. The predictions for the total concentrations of the chemical mixtures were very good especially for the PAH and elements including Pb, Al, Cr, Cd, Fe, Ni, and Zn; good to fair for the PHC, As and Se and fair to poor for the ALK and Cu. In contrast the predictions of the bioavailable concentrations of both PHC and HM were generally weaker than the total concentrations probably due to the small data set used for the calibration and prediction and overall lower concentrations values (≤50% of the total concentration value). Nevertheless, the results are promising and better than other studies focusing only on total concentrations. Overall this study confirmed that coupling VIS-NIRS to machine learning model offers a promising way forward to speed-up site investigation, identify and discriminate contaminant (i.e. hydrocarbons vs heavy metals) and predict not only the total concentration of the chemical of concern but also the concentration likely to pose significant risk (bioavailable) and therefore inform the risk assessment and decision making for contaminated sites in a timely fashion.

## Supplementary information

Supplementary materials

## Data Availability

All data generated or analysed during this study are included in this published article (and its Supplementary Information files).
