# Supplementary material for: Predicting bioavailability change of complex chemical mixtures in contaminated soils using visible and near-infrared spectroscopy and random forest regression
Source: Sci Rep. 2019 Mar 14;9:4492. doi: 10.1038/s41598-019-41161-w (PMC6418180; doi:10.1038/s41598-019-41161-w)
Supplement: Supplementary file 1 — Supplementary materials [file 41598_2019_41161_MOESM1_ESM.pdf]

Supplementary information - Predicting bioavailability change of complex chemical mixtures in contaminated soils using visible and near-infrared spectroscopy and random forest regression

Cipullo S. [1], Nawar S. [2], Mouazen A.M.[2], Campo-Moreno P. [1], Coulon F. [1*]

[1]Cranfield University, School of Water, Energy and Environment, Cranfield, MK430AL, UK

[2]Department of Environment, Ghent University, Coupure 653, 9000 Gent, Belgium

*Corresponding author: f.coulon@cranfield.ac.uk, +44 (0)1234 754981

Soils physicochemical characteristics

All soil samples physicochemical properties are summarised in Table S1. The samples collected at industrial sites, Soils 1, 2, and 3 were found to have low total nitrogen (700, 800 and 1200 mg/kg) and phosphorus (450, 430 and 500 mg/kg) contents, as well as, very alkaline pH (above pH 9). The high C/N ratio recorded for heavily polluted soils, indicates the presence of carbon molecules from the large amount of hydrocarbons present in these samples. The samples collected at the rural sites (Soil 4 and 5) had a clay loam texture (sand content < 35%), a neutral to alkaline pH (7.0 - 8.0), and an overall higher nitrogen and phosphorous contents.

Table S1: Physico-chemical properties of the five soil samples used in the study.

		Industrial			Rural	
**Characteristics**	**Analysis**	Soil 1	Soil 2	Soil 3	Soil 4	Soil 5
**Nutrients**	Total N (%)	0.07	0.08	0.12	0.23	0.25
	Total C (%)	4.00	4.14	3.87	2.39	2.78
	C:N	57.28	52.86	31.71	10.44	10.93
	Total P (mg/kg)	453.37	433.73	499.60	798.59	801.12
	Available P (mg/kg)	31.55	30.55	42.18	35.22	36.72
**Physical properties**	70% of WHC_{max} (% m/m)	21.92	20.37	19.64	39.21	40.02
	20% of WHC_{max} (% m/m)	6.26	5.99	5.61	11.20	12.00
	Dry matter content W_{dm} (%)	78.40	76.37	79.88	68.24	68.28
	Water content (%)	27.55	30.94	25.19	46.53	46.46
**Chemical properties**	pH	9.71	9.56	9.22	7.99	7.54
	LOI (%)	4.28	3.97	5.44	5.99	6.49
**Stone/gravel content**	% > 5.5 mm	25.97	23.47	20.13	0.00	0.00
	% 5.5 mm< > 2 mm	24.54	27.00	38.73	24.69	23.57
	% < 2 mm	49.48	49.54	41.15	75.31	76.43
**Particle size**	% 0.6 - 2 mm (Coarse sand)	11.88	13.65	16.86	3.55	4.36
	% 0.2 – 0.6 mm (Medium sand)	29.86	33.41	34.58	14.90	14.46
	% 0.06 - 0.2 mm (Fine sand)	30.37	27.04	20.24	11.70	11.29
	Overall sand content	72.11	74.10	71.68	30.16	30.10
	% 0.002 mm - 0.06 mm (Silt)	19.67	16.70	16.14	40.57	36.07
	% < 0.002 mm (Clay)	8.22	9.20	12.17	29.28	33.83

N: nitrogen; C: carbon; P: phosphorous, WHC: water holding capacity; LOI: loss of ignition.

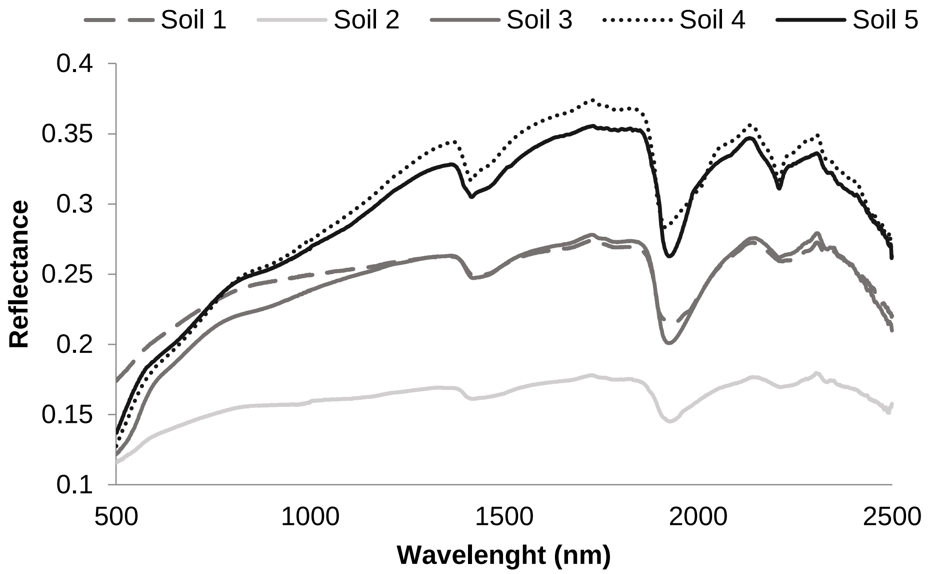

Figure S1: Average VisNIR diffuse reflectance spectra of the five soil samples analysed.

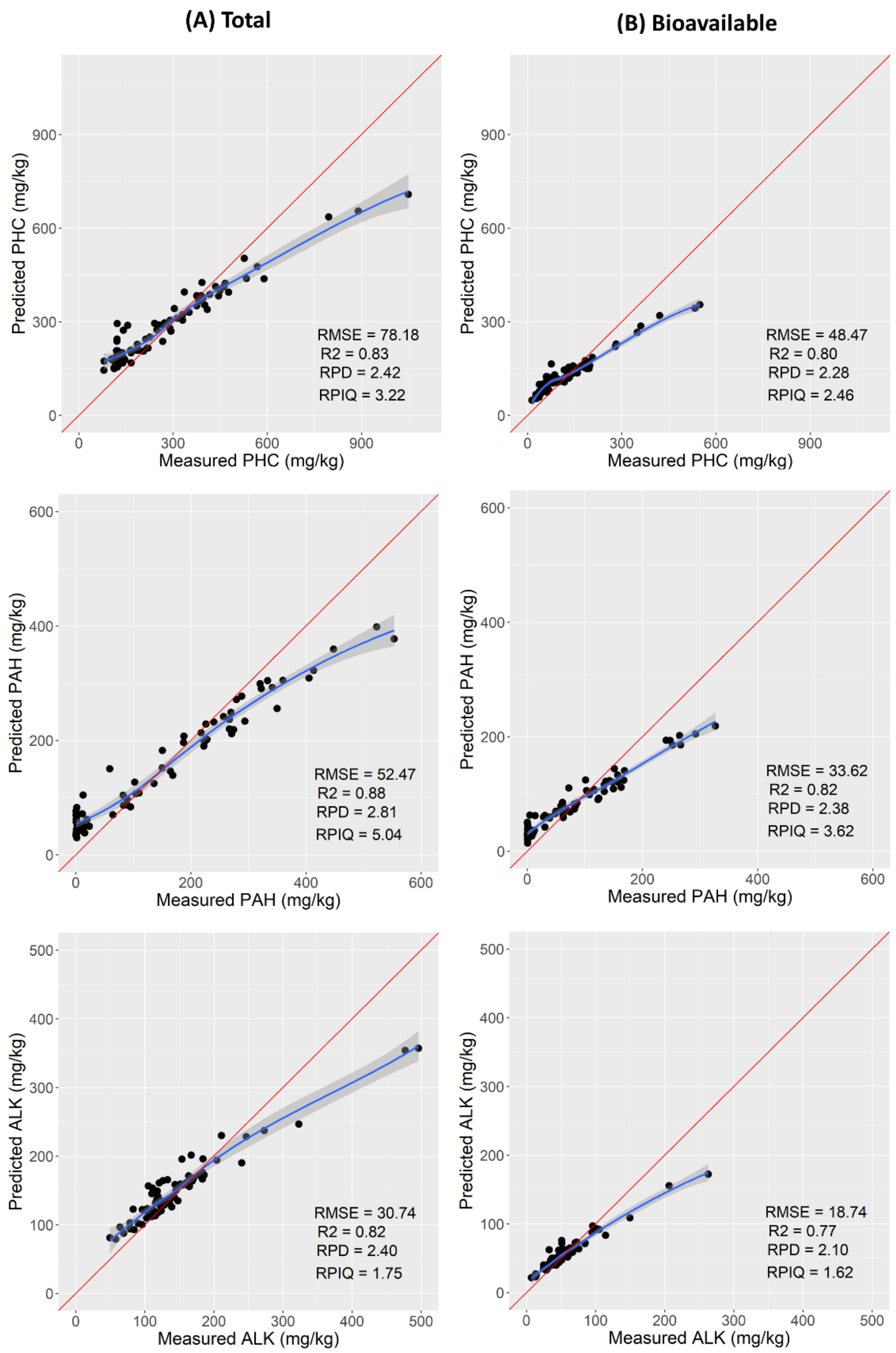

Figure S2: Scatter plots of the calibration datasets of total (a) and bioavailable (b) contents of petroleum hydrocarbon (PHC), polycyclic aromatic hydrocarbon (PAH), alkanes (ALK).

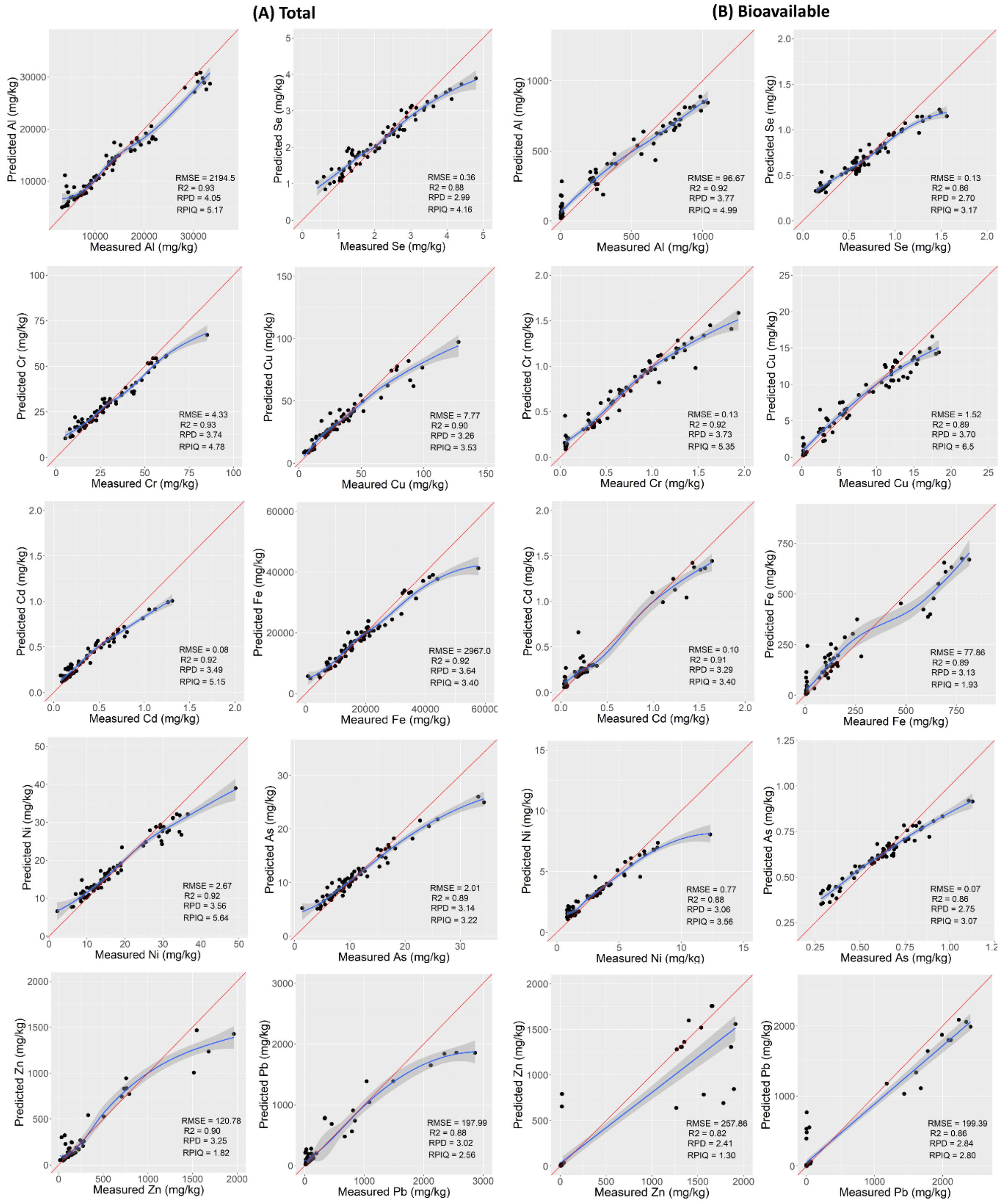

Figure S3: Scatter plots of the calibration datasets of total (a) and bioavailable (b) contents of HM/metalloids (Al, Cr, Cd, Ni, Zn, Se, Cu, Fe, As, and Pb).
